# Nasopalatine duct cyst: a multicenter retrospective study of 63 cases and an integrative review of the clinical, imaginological and histopathological aspects

**DOI:** 10.1590/1678-7757-2024-0539

**Published:** 2025-05-02

**Authors:** Diana Estefania RAMOS PEÑA, Mariana de Sá ALVES, Samuel Porfírio XAVIER, Tiago Novaes PINHEIRO, Paulo Sérgio da Silva SANTOS, Izabel Regina Fischer RUBIRA-BULLEN, Ana María CADAVID GIRALDO, Pilar Schmitt SANJUAN NAVARRO, Leonor Victoria GONZÁLEZ-PÉREZ, Cintia Micaela CHAMORRO PETRANACCI, Mario PEREZ-SAYÁNS, Ana Lia ANBINDER, Janete Dias ALMEIDA, Ana Carolina Fragoso MOTTA

**Affiliations:** 1 Universidade de São Paulo Faculdade de Odontologia Departamento de Estomatologia São Paulo São Paulo Brasil Universidade de São Paulo, Faculdade de Odontologia, Departamento de Estomatologia, São Paulo, São Paulo, Brasil.; 2 Universidade Estadual Paulista Instituto de Ciência e Tecnologia Departamento de Biociências e Diagnóstico Bucal São José dos Campos São Paulo Brasil Universidade Estadual Paulista (UNESP), Instituto de Ciência e Tecnologia, Campus de São José dos Campos, Departamento de Biociências e Diagnóstico Bucal, São José dos Campos, São Paulo, Brasil.; 3 Universidade de São Paulo Faculdade de Odontologia de Ribeirão Preto Departamento de Cirurgia e Traumatologia Buco-Maxilo Facial e Periodontia Ribeirão Preto SP Brasil Universidade de São Paulo, Faculdade de Odontologia de Ribeirão Preto, Departamento de Cirurgia e Traumatologia Buco-Maxilo Facial e Periodontia, Ribeirão Preto, SP, Brasil.; 4 Universidade do Estado do Amazonas Divisão de Estomatologia e Patologia Oral Manaus Amazonas Brasil Universidade do Estado do Amazonas, Divisão de Estomatologia e Patologia Oral, Manaus, Amazonas, Brasil.; 5 Universidade de São Paulo Faculdade de Odontologia de Bauru Departamento de Cirurgia, Estomatologia, Patologia e Radiologia Bauru São Paulo Brasil Universidade de São Paulo, Faculdade de Odontologia de Bauru, Departamento de Cirurgia, Estomatologia, Patologia e Radiologia, Bauru, São Paulo, Brasil.; 6 Universidad de Antioquia Facultad de Odontología Medellín Colombia Universidad de Antioquia, Facultad de Odontología, Grupo de investigación en Patología Oral, Periodoncia y Cirurgía-Alveólo-Dentaria – POPCAD, Medellín, Colombia.; 7 Faculty of Medicine and Dentistry Universidade de Santiago de Compostela Santiago de Compostela España Oral Medicine, Oral Surgery and Implantology Unit (MedOralRes). Faculty of Medicine and Dentistry Universidade de Santiago de Compostela, Santiago de Compostela, España; 8 Instituto de Investigación Sanitaria de Santiago Santiago de Compostela España Instituto de Investigación Sanitaria de Santiago (IDIS), ORALRES group Santiago de Compostela, Santiago de Compostela, España.; 9 Instituto de los Materiales de Santiago de Compostela Santiago de Compostela España Instituto de los Materiales de Santiago de Compostela (iMATUS), Santiago de Compostela, España.; 10 Universidade de São Paulo Faculdade de Odontologia de Ribeirão Preto Departamento de Estomatologia, Saúde Coletiva e Odontologia Legal Ribeirão Preto São Paulo Brasil Universidade de São Paulo, Faculdade de Odontologia de Ribeirão Preto, Departamento de Estomatologia, Saúde Coletiva e Odontologia Legal, Ribeirão Preto, São Paulo, Brasil.

**Keywords:** Nonodontogenic cysts, Diagnostic imaging, Oral Pathology, Oral diagnosis, Multicenter Study

## Abstract

The nasopalatine duct cyst (NPDC) is the most common nonodontogenic jaw cyst, with a higher prevalence reported in males. Diagnosing jaw cysts remains challenging for general dentists due to their overlapping clinical and radiographic presentations. An updated case series and integrative literature review may assist in improving the accurate diagnosis of NPDC.

**Objective:**

This study aimed to describe the clinicopathological and imaging characteristics of 63 NPDC cases and to review previously reported cases in the literature.

**Methodology:**

An international, multicenter, retrospective NPDC case series was conducted. Demographic, radiographic, and histopathological data were collected from clinical records. Additionally, a PubMed/MEDLINE search was performed to identify articles on NPDC.

**Results:**

A total of 63 NPDC cases were evaluated, with a mean patient age of 47 years and no significant sex predilection. Twenty-one cases were asymptomatic, while 34 presented with symptoms such as pain and swelling. Radiographically, NPDC appeared as a well-defined radiolucent lesion located between the upper central incisors, bordered by a radiopaque margin. The integrative literature review identified 67 studies, comprising 51 case reports, 12 retrospective studies, and four case series, totaling 1,003 reported NPDC cases. The clinicopathological and radiographic findings from the literature aligned with those in this case series.

**Conclusion:**

The 63 cases analyzed in this study showed consistent findings across six international centers, with no sex predilection observed, contrasting with the male dominance reported in the literature. NPDC should be considered in the differential diagnosis of intraosseous lesions in the anterior maxilla. Accurate diagnosis requires a combination of radiographic and histopathological evaluations to prevent misdiagnosis and improper treatment.

## Introduction

Nasopalatine duct cyst (NPDC), also known as incisive canal cyst, is a fissural cyst that was first described as a paranasal sinus.^1^ It is the most common nonodontogenic jaw cyst, with a reported prevalence ranging from 2.2% to 32.8% and a higher incidence in males between the fourth and sixth decades of life.^2-4^ The widely accepted origin of NPDC is the proliferation of epithelial remnants from the embryologic nasopalatine duct.⁵ Additionally, localized trauma, infections, and irritations have been suggested as potential triggers for the proliferation of epithelial cell remnants within the nasopalatine duct. Spontaneous proliferation has also been proposed.⁶^,^⁷

Asymptomatic NPDC can be detected by routine imaging examinations, mimicking a periapical lesion of endodontic origin.^8^It develops in the anterior maxilla and appears as a heart-shaped configuration on periapical radiographs due to superposition of the anterior nasal spine or the nasal septum. On maxillary occlusal radiographs, asymptomatic NPDC may appear as a round or ovoid radiolucency near or close to the midline between the upper central incisors.^9^ In most cases, the involved teeth had a positive reaction to pulp sensitivity testing.^10^

Despite the characteristic features and high prevalence of NPDC, there remains considerable variability in the reported clinical, radiographic, and histopathological characteristics, as well as in its demographic distribution. Misinterpretation and misdiagnosis still occur; in some cases, NPDC is clinically mistaken for a periapical lesion, potentially leading to inappropriate endodontic treatment or even unnecessary tooth extraction, without lesion resolution.⁸^,1 1^ A major gap in the current literature is the limited availability of large, multicenter studies evaluating NPDCs with a standardized approach to clinical and histopathological assessments. Most existing studies focus on isolated case reports or small case series, making it difficult to establish comprehensive epidemiological patterns or assess potential regional differences in presentation. Additionally, despite the histological features of NPDCs being well-described, there is little data correlating clinicopathologic variables across different diagnostic centers, which could provide valuable insights into potential diagnostic discrepancies or variations in disease presentation. Furthermore, while several reviews of NPDCs exist, an updated literature review incorporating recent data is lacking. Therefore, this multicenter retrospective study aimed to describe the clinical, radiographic, and histopathological features of 63 NPDC cases diagnosed at six oral medicine centers. Additionally, we conducted an integrative review of the literature and compared the key characteristics of NPDC reported in the literature with those observed in our cases.

## Methodology

### Retrospective case series collection

**Figure 1:**
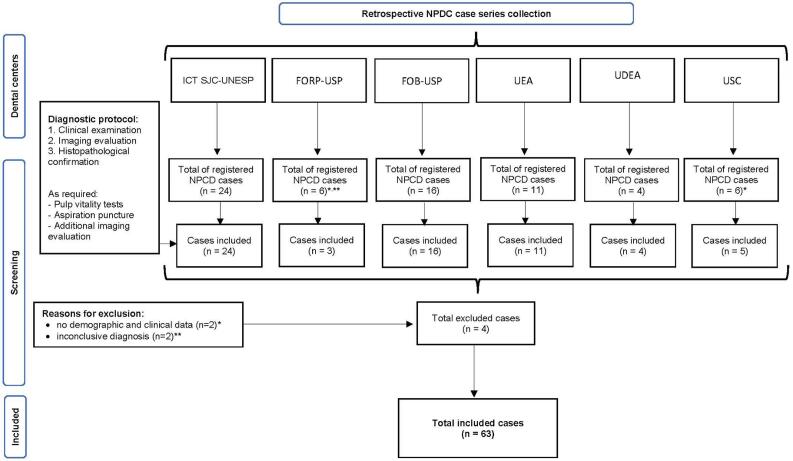
Flow diagram of the inclusion criteria and standardized diagnostic protocol applied in the six centers

A retrospective case series of nasopalatine duct cyst (NPDC) cases was conducted at six dental centers in different countries to gather data reflecting a wide array of clinical presentations. The institutions included: Institute of Science and Technology, São Paulo State University (ICT SJC-UNESP), São José dos Campos, SP, Brazil (n=24); School of Dentistry of Ribeirão Preto, University of São Paulo (FORP-USP), Ribeirão Preto, SP, Brazil (n=3); School of Dentistry of Bauru, University of São Paulo (FOB-USP), Bauru, SP, Brazil (n=16); Amazonas State University (UEA), Manaus, AM, Brazil (n=11); Facultad de Odontología, Universidad de Antioquia (UDEA), Medellín, Colombia (n=4); Faculty of Medicine and Odontology, University of Santiago de Compostela (USC), Santiago de Compostela, Spain (n=5) (Figure 1). Each NPDC case included demographic and clinical data, such as age, sex, race, lesion location, duration, size, imaging and histopathologic features, local signs, symptoms, and treatments administered, capturing the diversity of presentations across these regions. The study was approved by the Research Ethics Committee of the involved institutions (Brazil—CAAE: 28751020.4.0000.5016; international centers: 2019/596).

### Inclusion criteria and standardized diagnostic protocol

In this multicenter retrospective study, NPDC cases were included based on specific criteria to ensure consistency and comparability across centers. The inclusion criteria required that all cases have complete clinical and radiographic data available for review, along with a confirmed histopathological diagnosis of NPDC following enucleation or marsupialization. A standardized diagnostic protocol was implemented across all participating centers. The diagnostic approach included a comprehensive clinical examination and radiographic evaluation, utilizing periapical radiographs, panoramic radiography, and/or cone-beam computed tomography (CBCT), followed by histopathological confirmation. The histopathological assessment was conducted by experienced oral pathologists at each center, adhering to internationally recognized diagnostic criteria. Additionally, pulp vitality tests were performed to differentiate NPDC from periapical lesions of endodontic origin. In cases in which the diagnosis remained uncertain, additional imaging studies or aspiration procedures were conducted to rule out other cystic or neoplastic lesions.

### Integrative review of the literature

An integrative review of NPDC literature complemented the case series analysis. This review searched PubMed/MEDLINE, Embase, and Scopus databases with the terms “nasopalatine duct cyst,” “NPDC,” “nonodontogenic cyst,” and “cysts of the jaws.” Articles were selected based on criteria that ensured comprehensive inclusion of demographic data (e.g., age, sex) and specific descriptions of NPDC cases, including clinical manifestations, radiographic findings, and histopathological features. Articles missing any key information on clinical or radiographic characteristics of NPDCs were excluded to maintain analytical consistency.

### Data analysis

Demographic and clinical data from both the case series and literature were then analyzed to determine patterns of NPDC presentations and treatment across the varied geographic sample. The results were expected to reveal similarities and differences in clinical features, diagnostic findings, and treatment approaches between different populations, potentially enhancing diagnostic understanding and standardization in NPDC management.

## Results

### Case series

**Figure 2:**
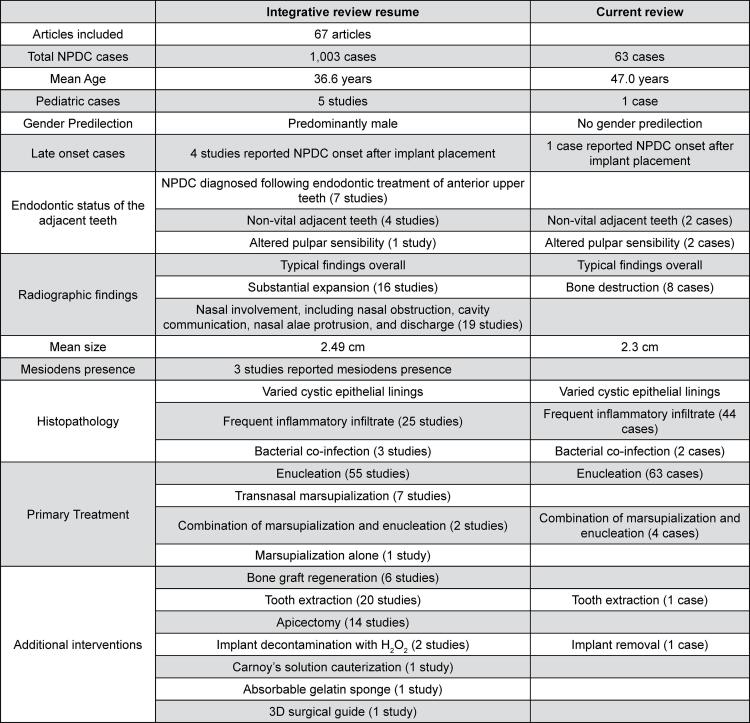
Summary of the descriptive characteristics of nasopalatine duct cyst reported in the articles included in the review (n = 67)

**Figure 3:**
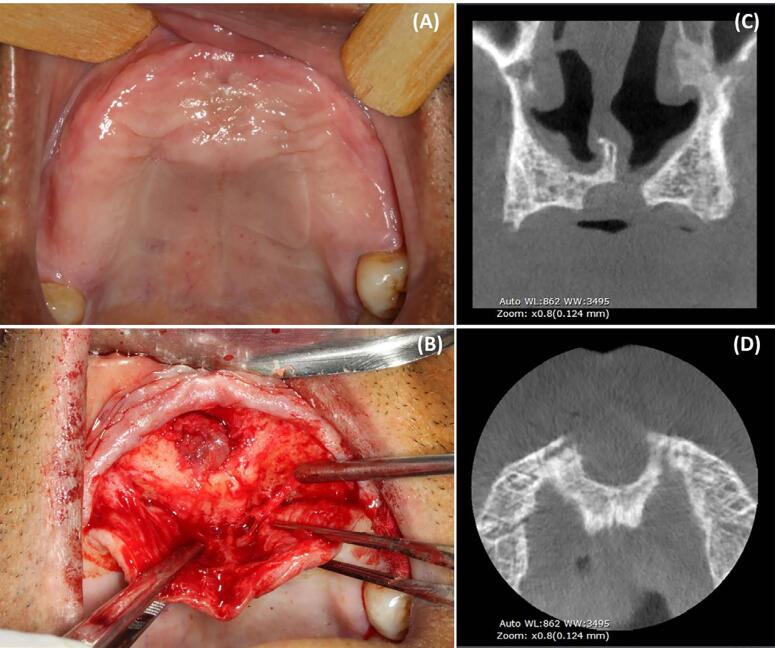
Clinical and imaging appearance of a nasopalatine duct cyst. a. Swelling along the midline of the anterior hard palate. b. Intraoperative view showing the cystic lesion. Coronal (c) and axial (d) images of cone-beam computed tomography showing a well-defined corticated hypodense cystic lesion

The clinical, radiographic and histopathological characteristics of each case are summarized in Figure 2 along with the reviewed literature; for details of each case see Supplementary Table 1. Of the 63 patients included in this study, 31 (49.2%) were women and 32 (50.8%) were men, with a mean age of 47.0 years (range 10-79 years). There was only one pediatric case of a ten-year-old girl. Regarding race, 39 patients (61.9%) were white, 15 (23.8%) were black, one (1.6%) was Asian, and three were of multiracial origin (4.8%). There was no information on race for five cases (7.9%). Thirty-four (53.9%) patients developed symptomatic clinical changes, including pain and swelling (Figure 3a), drainage was present in 10 cases (15.9%), 21 (33.3%) cases were completely asymptomatic, six (9.52%) presented teeth displacement or mobility. Other signs and symptoms present in five cases (7.9%) included discomfort, halitosis, and dental sensitivity; no information was available for three cases (4.7%).

The radiographic findings were typical of NPDC and included a homogeneous well-defined radiolucent lesion with an oval or heart-like shape, which was generally located between the upper central incisors, bound by a radiopaque line. Cone-beam computed tomography (CBCT) was performed in 15 cases, showing a hypodense circumscribed lesion at the level of the nasopalatine duct or at the level of the apex of the upper incisors. Eighteen cases presented some form of bone destruction, and only one case presented root resorption. The CBCT axial and coronal images of a NPDC case shown in Figure 3c and 3d, respectively, revealed a hypodense round lesion with well-defined limits.

An initial clinical diagnosis of NPDC was given in 29 of the 63 cases and was considered a secondary diagnosis in 10 cases. Residual cyst, inflammatory odontogenic cyst, traumatic bone cyst and keratocyst were considered differential diagnosis. In 20 cases a different clinical diagnosis from NPDC was given, including odontogenic cysts, residual cyst, periapical cyst, simple bone cyst, odontogenic tumor, keratocyst, and mucous retention phenomena.

Aspiration puncture was performed as a complementary exam before surgical treatment in four cases; liquid bloody content was identified in two cases and the other two cases presented clear yellowish and bloody fluid. A dental implant associated with the cyst was present in one case, which was removed during surgical treatment. Enucleation was performed in all cases (Figure 3b). In four cases of large cysts, marsupialization was performed before enucleation. Extraction of the involved teeth was described in only one case.

**Figure 4:**
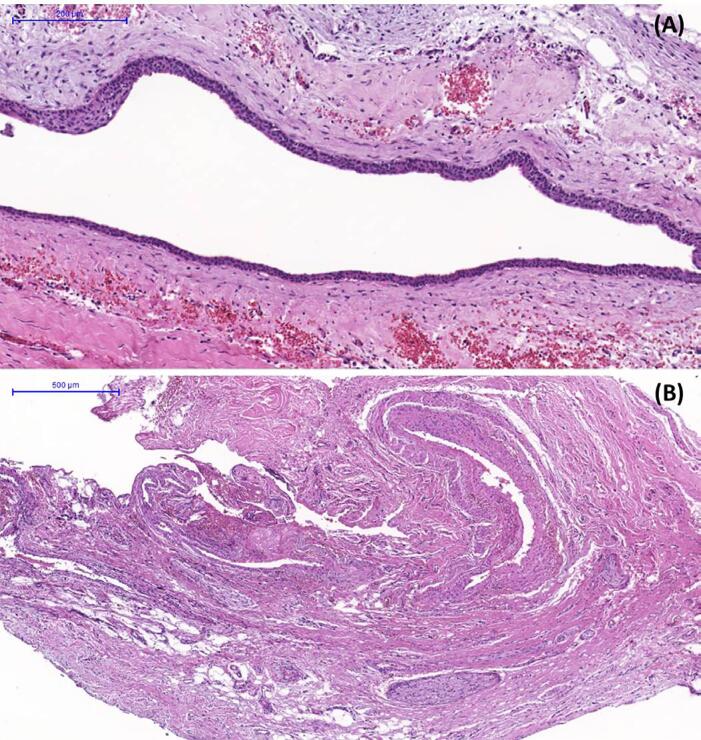
Histological appearance of a nasopalatine duct cyst. a. The cystic capsule is lined by thin non-keratinized stratified squamous or cuboidal epithelium. Hematoxylin and eosin staining. Scale bar = 200 µm. b. Neurovascular bundles are present in the cystic capsule. Hematoxylin and eosin staining. Scale bar = 500 µm. All slides were digitized using a whole slide scanner (Pannoramic Desk, 3DHistech), with a x20 objective

Histological examination was performed in all cases and revealed fragments of the cyst wall lined by epithelium of variable architectural patterns, including combinations of cuboidal, pseudostratified columnar and squamous epithelia, all of them non-keratinized (Figure 4a). The connective tissue of the capsule was dense, with the observation of chronic inflammation, nerve bundles and muscular arteries in the majority of cases (Figure 4b). Bacterial colonies were present in two cases and Actinomyces sp. was identified in one case. Salivary gland structures and neurovascular bundles were also observed in several cases.

### Literature review

**Figure 5:**
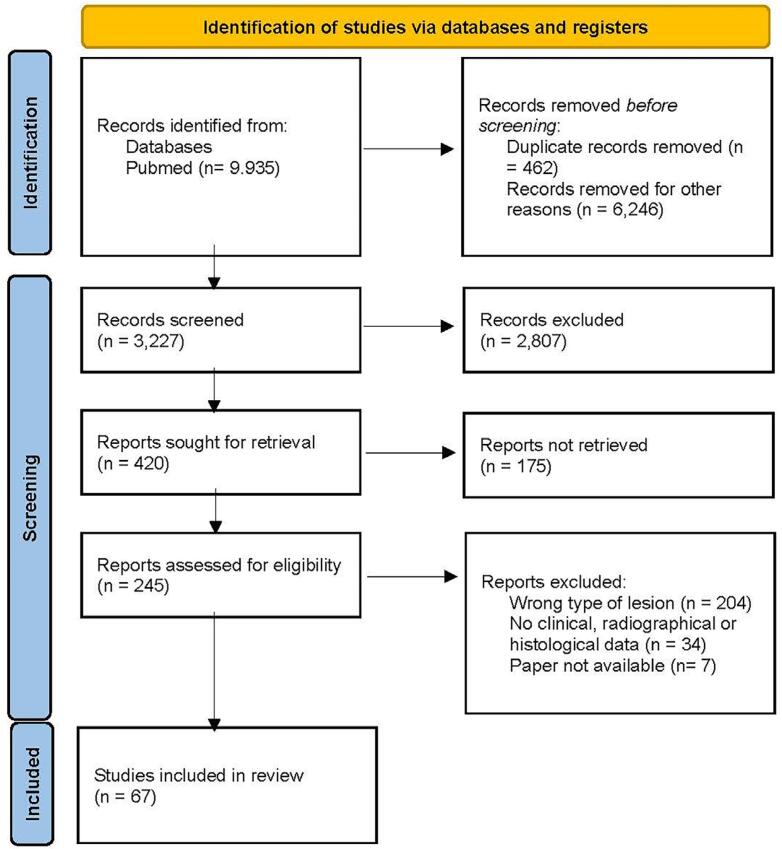
Study selection process according to PRISMA 2020 flow diagram12

Of 9,935 results identified in the PubMed/MEDLINE database, 461 articles were screened, and 67 articles were included for review, comprising a majority of case reports (51 studies), followed by 12 retrospective studies and four case series (Figure 5). A total of 1003 cases were reported in the 67 articles. The mean age in the reviewed studies was 36.6 years and there was male predilection. Regarding local signs and symptoms, swelling in the anterior maxilla was frequently observed across many studies, often accompanied by pain.^5,8^ Other symptoms include bluish discoloration,^14,39,43,46,54^ drainage and/or sinus tract formation,^5,9,11,13,19,23,38,40,46,55^ discomfort^50,57^ and tooth mobility;^23,28^ some cases were asymptomatic and discovered the cyst incidentally.^5,7,9,15,40,47,60^ Overall, the radiographic findings described a round, oval, pear-shaped or heart-shaped well-circumscribed radiolucency. In some cases, radiographic images showed displacement of the upper anterior teeth, cortical bone destruction, and expansion into the nasal cavity.^8,16^ CT scans were reported in 28 studies and MRI in two studies, with the first CT scan used for the diagnosis of NPDC reported by Hertzanu, Cohen and Mendelsohn^32^ (1985). Histopathological data reported the presence of a variety of epithelial linings, with many cases exhibiting mixed epithelial linings with transitions between types, including stratified squamous epithelium, cuboidal and columnar epithelium, often ciliated and pseudostratified ciliated columnar epithelium, also known as respiratory epithelium. Chronic inflammatory infiltrate was often noted in the cystic capsule. Neurovascular bundles were commonly present in the cyst wall, along with minor salivary glands in some cases. Hemorrhage, collagen fiber bundles and mucous glands were displayed in the fibrous capsule in some cases. Additionally, some variations of the surgical approach were reported including bone graft regeneration in the bone defect area,^8,11,14,27,34,35^ extraction of the involved teeth,^18^ apicectomy,^14^ cauterization with Carnoy’s solution,^29^ implant decontamination with H2O2,^27,34^ placement of absorbable gelatin sponge^28^ and the use of a 3D surgical guide.^60^ Table 1 shows a summarized comparison between the present study and the included articles (n=67) on NPDC (for more details see Supplementary Table 2).

## Discussion

In this NPDC case series, there was no sex predilection, which is in accord with other retrospective studies of the literature.^16,47,50,69^ Among the reviewed studies a slight, ^40^ or significant^5,7,9,10,15,20,36,46,49,70,71^ male predilection between case series and retrospective studies was found, with 38 case reports of male patients.^8,11,13,14,17,19,21,23^ There were five NPDC case reports in pediatric patients.^28,41,43,54,56^ Although information regarding race was not provided in the majority of the reviewed studies, some studies reported increased prevalence in White patients,^9,38,40,50^ with only one study reporting a more aggressive presentation in Black patients.^36^ One third of our cases were asymptomatic; clinical sign and symptoms included pain, drainage and the presence of a fistula; these symptoms can be the result of a secondary infection of the cyst.^8,10^ Large cysts located in the upper part of the duct can also cause nasal obstruction and bone fenestration,^8,16^as seen in two of our cases. Edentulous patients also reported ill-fitting dentures. Local inflammation and ulceration caused by dentures have also been reported in previous retrospective studies.^8,47^

We identified the presence of bacterial colonies in two cases. It should be noted that one case showed a significant swelling in the anterior maxilla accompanied by drainage and an ill-fitting denture; Actinomyces spp. was identified based on the presence of sulfur deposits inside the bacterial agglomerates. These bacteria normally inhabit the mouth and upper respiratory tract,^72^ and disruption of the mucosal barrier by trauma, surgery or preceding infection can lead to bacterial invasion of the adjacent tissues, causing inflammation.^72,73^ Most cases of actinomycosis that occur in the head and neck are associated with poor oral hygiene, invasive dental procedures or oral trauma.^74^ Only two cases of Actinomyces spp infection associated to NPDC were found in the literature.^33,75^ Additionally, one case of Staphylococcus epidermidis co-infection was also reported by Kim, Moon and Lee^26^ (2023). Secondary infection of NPDC can be associated with the presence of a patent nasopalatine duct canal, which creates eventual communication between the oral cavity and the nasopalatine duct. Once present, this communication could favor NPDC contamination by Actinomyces spp. and S. epidermidis from the oral cavity.

NPDC is diagnosed as an inflammatory odontogenic lesion in up to 11.46% of the cases;^77^ a recent systematic review pointed NPDC as the fourth most common lesion that mimics endodontic lesions,^76^ which can lead to misdiagnosis and inappropriate treatment.^11,59,77^ The correct diagnosis should be based on positive pulp vitality testing and negative percussion tests, however, as shown in the present review, some cases can present negative vitality test due to the aggressive nature of the cyst.^15,28,29,71^

Large NPDC pose a diagnostic challenge since they are difficult to differentiate from periapical cysts related to the incisive teeth, which may present pulp vitality tests that are difficult to interpret. In these cases, radiographic evaluation of the teeth adjacent to or associated with a cyst-like lesion is essential for diagnosis.^47,77^ In the present integrative literature review, eight studies reported endodontic treatment prior to the diagnosis of NPDC, mainly due to a misdiagnosis of the cyst as a periapical lesion of endodontic origin.^8,11,13^ In three studies,^8,11,34^ the teeth were extracted because of the persistence of the lesion after endodontic treatment. Moreover, one study reported a history of transoral sublabial excision performed twice in an attempt to drain an NPDC misdiagnosed as an acute dentoalveolar abscess.^19^ The number of cases of mistreatment of NPDC may be higher than the rates found in the literature since the reason for the endodontic or extraction treatment is not reported in several case reports and retrospective studies.

McCrea^14^ (2014) and Alassaf, et al.^25^ (2023) reported interesting cases in which two synchronous diagnoses were made regarding the same patients: a periapical granuloma accompanying a NPDC, and a radicular cyst simultaneous to a NPDC, respectively. In these cases, both enucleation and apicectomy were performed.^14,25^ A similar case is reported in our case series, in which a final diagnosis of NPDC associated with a periapical cyst was concluded. Other conditions should be included in the differential diagnosis, such as enlarged nasopalatine ducts, central giant cell granulomas, supernumerary teeth, follicular cysts, osteitis fistulizing in the palatine direction, or bucconasal and/or buccosinusal communication.^15,47^ In cases of edentulism, the residual cyst should be included as a differential diagnosis.^8^

In one of our cases, NPDC was associated with an implant that had to be removed during surgical treatment of the cyst. McCrea^14^(2014) and Casado et al.^34^ (2008) also reported the presence of NPDC associated with implants in the upper incisors area. In these two cases a periapical cyst was diagnosed before tooth extraction; however, recurrence of the bone lesion occurred after implant placement. On the other hand, two recent case reports described satisfactory results with implant surface decontamination using H2O2 along with bone regeneration, which avoided the loss of the implants, generally associated to NPDC.^27,35^ The description of these five cases highlights the importance of histopathological analysis, since the symptoms of periapical implant lesions or apical peri-implantitis in the anterior region can be similar to those of NPDC.^78^ In cases in which upper anterior implants are present, periapical implant lesions should be included in the differential diagnosis and histopathological analysis is necessary for an adequate diagnosis and treatment.

NPDC diagnosis should be based on the site of the lesion (upper central incisors or anterior maxilla), radiographic and histological features. Histology is considered the gold standard for the diagnosis of NPDC.^69,79^ The clinicopathological features of NPDC have been documented since the first case described by Meyer^1^ in 1914. However, due to its histological diversity, the histopathogenesis of NPDC remains controversial.^1,71^ The predominant epithelial lining of these cystic lesions is stratified squamous epithelium alone or combined with another type,^69^ which can be related to the proximity to the nasal cavity.^16^ Cysts located superiorly are lined by respiratory epithelium, while those located closer to the oral cavity are lined by stratified squamous epithelium.^7^ Histopathological examination of our case series revealed multiple epithelial types, including stratified, pseudostratified and simple non-keratinized epithelium, being columnar, cuboidal and squamous the most common epithelial types. The variation in the epithelial lining of the NPDC may lead to a misinterpretation by less experienced pathologists and could be confused with glandular odontogenic cysts. Epithelium compatible with the respiratory type was present in 23 cases and nine cases exhibited hyperplastic areas. Mucous salivary glands were observed in 17 cases. Inflammatory infiltrate was prevalent among our cases and was observed in 44 out of the 63 cases. In addition to histopathological examination, immunohistochemistry can contribute to the diagnosis of NPDC, confirming its lining with stratified squamous epithelium.^47^

Radiological exploration is essential for the diagnosis of NPDC, and new methods to obtain high NPDC detection in panoramic radiography are being explored, such as deep convolutional neural networks, an algorithm that is able to classify and detect lesions using images.^80^ In addition to panoramic radiography, other techniques are recommended, such as periapical and occlusal radiographs.^10^ Despite the possibility of the nasopalatine duct cyst presenting unusual radiographic characteristics, in most of our cases, panoramic radiography showed a round, radiolucent, well-circumscribed image, delimited by a sclerotic margin. In the cases in which CBCT was performed, a hypodense round image was identified in the incisive canal, as well as cortical bone fenestrations. In the presence of expanding lesions, the use of CBCT or magnetic resonance imaging might be required to determine the exact position and define the best surgical approach.^15,81^ Suter, et al.^49^ evaluated the dimensions of NPDC using standardized CBCT protocols and concluded that the risk of postoperative complications increases with the size of the cyst. Another study by the same team^81^ reported that bulging signs (local enlargement of the canal) can suggest an early stage of NPDC, and inflammatory processes in the neighboring teeth should be identified and eliminated since they may induce bulging of the nasopalatine canal and/or the formation of NPDC. An enlarged nasopalatine duct is one of the most pertinent differential diagnoses for NPDC.^82^ Since CBCT enables 3D analysis, which favors diagnostic findings, a cut-off value of 10 mm has been developed to create clearly defined differential diagnosis parameters.^83^ Regarding the differential diagnosis for further lesions in the anterior region of the hard palate, CBCT will support the diagnosis by establishing the connection of the lesion to the nasopalatine duct.

Although NPDC has been described as a self-limited expansive lesion, an aggressive growth potential has been reported in the presence of bone dehiscence.^14,17,28,31^Surgical enucleation is the preferred treatment approach for NPDC, offering a high success rate and minimal risk of recurrence. Marsupialization may be considered for large cysts or for cases in which the location of the cyst poses challenges.^16^ In this case series, the lesions were completely removed by enucleation. Only three cases required marsupialization before enucleation. In cases in which resorption of the nasal floor is present, transnasal access to the lesion is recommended, as described in seven reviewed studies.^16,19,20,22,26,30,58^The clinical prescription of dental radiographs should be based on professional judgment,^84^ and considering the importance of the earlier detection for more conservative treatment, we would suggest dental radiographs once a year even for asymptomatic cases.

This study presents some limitations, inherent to the retrospective nature of the study, including the lack of information on the race of the patients, postoperative evolution of the cases, and information about the size of the lesion, since data from only 12 cases was retrieved. This lack of information limits the projection of our results to broader populations. In view of the potential bias related to geographic and institutional centers, the cases included in this study were selected based on the same histopathological analysis and diagnosis. Despite this, we present a substantial number of cases of six different centers, which showed similar clinical, radiographical and histopathological characteristics. Overall, our results were in accordance with the NPDC features found in the literature review, except for sex occurrence, since no sex predilection for NPDC was observed, while a higher male prevalence was found in the literature review. In addition, a lack of substantial case series and retrospective NPDC studies in the last three years was observed. In conclusion, NPDC is a common nonodontogenic cyst that can be easily misdiagnosed and mistreated. We recommend that the diagnostic hypothesis of NPDC should be included in all cases of intraosseous lesions in the median anterior maxillary. For clinical practice, we suggest incorporating radiographic and CBCT evaluations as part of the initial diagnostic workflow to identify potential NPDCs early. Histopathological analysis should be performed to confirm the diagnosis and guide appropriate treatment, thereby preventing complications and ensuring correct management. Given the lack of substantial case series and retrospective studies in the last few years, further research is needed to establish more comprehensive diagnostic protocols for NPDC.
